# Development of an ultra-high sensitive immunoassay with plasma biomarker for differentiating Parkinson disease dementia from Parkinson disease using antibody functionalized magnetic nanoparticles

**DOI:** 10.1186/s12951-016-0198-5

**Published:** 2016-06-08

**Authors:** Shieh-Yueh Yang, Ming-Jang Chiu, Chin-Hsien Lin, Herng-Er Horng, Che-Chuan Yang, Jen-Jie Chieh, Hsin-Hsien Chen, Bing-Hsien Liu

**Affiliations:** MagQu Co., Ltd., Xindian District, New Taipei City, 231 Taiwan; Institute of Electro-optical Science and Technology, National Taiwan Normal University, Taipei, 116 Taiwan; Department of Neurology, National Taiwan University Hospital, College of Medicine, National Taiwan University, Taipei, 100 Taiwan; Graduate Institute of Brain and Mind Sciences, College of Medicine, National Taiwan University, Taipei, 100 Taiwan; Department of Psychology, National Taiwan University, Taipei, 100 Taiwan; Graduate Institute of Biomedical Engineering and Bioinformatics, National Taiwan University, Taipei, 116 Taiwan

**Keywords:** α-synuclein, Parkinson disease, Immunomagnetic reduction

## Abstract

**Background:**

It is difficult to discriminate healthy subjects and patients with Parkinson disease (PD) or Parkinson disease dementia (PDD) by assaying plasma α-synuclein because the concentrations of circulating α-synuclein in the blood are almost the same as the low-detection limit using current immunoassays, such as enzyme-linked immunosorbent assay. In this work, an ultra-sensitive immunoassay utilizing immunomagnetic reduction (IMR) is developed. The reagent for IMR consists of magnetic nanoparticles functionalized with antibodies against α-synuclein and dispersed in pH-7.2 phosphate-buffered saline. A high-T_c_ superconducting-quantum-interference-device (SQUID) alternative-current magnetosusceptometer is used to measure the IMR signal of the reagent due to the association between magnetic nanoparticles and α-synuclein molecules.

**Results:**

According to the experimental α-synuclein concentration dependent IMR signal, the low-detection limit is 0.3 fg/ml and the dynamic range is 310 pg/ml. The preliminary results show the plasma α-synuclein for PD patients distributes from 6 to 30 fg/ml. For PDD patients, the concentration of plasma α-synuclein varies from 0.1 to 100 pg/ml. Whereas the concentration of plasma α-synuclein for healthy subjects is significantly lower than that of PD patients.

**Conclusions:**

The ultra-sensitive IMR by utilizing antibody-functionalized magnetic nanoparticles and high-T_c_ SQUID magnetometer is promising as a method to assay plasma α-synuclein, which is a potential biomarker for discriminating patients with PD or PDD.

## Background

Parkinson disease (PD) is the second most common neurodegenerative disease after Alzheimer’s disease. More than 1 % of people older than 65 years old are suffering from PD [1]. About 10 million people worldwide are living with PD. The direct and indirect healthcare cost for one PD patient is estimated to be US 100,000 per year [2]. Many countries, especially the US, Canada, Europe and Australia, are worrying about unsustainable increases in the costs of healthcare. Lots of resources and effort have been put into developing the diagnosis, treatments and vaccine for PD.

The clinical criteria for diagnosing PD are the observations of movement disorders such as bradykinesia, cogwheel rigidity, resting tremor and postural instability. Although these clinical features are popularly used, there are several fatal issues for diagnosing PD. For example, other movement disorders (e.g. multiple system atrophy, corticobasal degeneration, or progressive supranuclear palsy) might overlap with the clinical symptoms of PD and decrease the accuracy of diagnosing PD [3]. In addition, it has been reported the clinical symptoms are present after degeneration of over 50 % of dopaminergic neurons in the basal ganglia, particularly in the substantia nigra [4]. The early-stage diagnosis of PD is very difficult, using observations of clinical movement disorders. Analysis of the genetic sequence seems a better method for early-stage diagnosis of PD [5–7]. Nevertheless, only 10 % of PD patients are hereditary. Ninety percent of PD patients are sporadic.

Development of cognitive impairment and dementia, referred as Parkinson disease dementia (PDD), is common in PD [8]. The prediction of development of dementia in PD is challenging and of significant impact in the field. Researchers are now trying to achieve bio-molecular diagnosis for differentiating PD from PDD. α-synuclein is the most recognized biomarker for PD or PDD [9, 10]. As α-synuclein molecules are phosphorylated, phosphor-α-synuclein molecules easily aggregate with one another to form Lewy body in the dopaminergic neurons [11, 12]. Dopaminergic neurons with Lewy bodies become degenerative and lose the ability to express dopamine. Neural cells in the motor cortex of the brain are damaged due to the lack of dopamine and movement disorders are stimulated.

Numerous discoveries show the concentration of α-synuclein in the cerebrospinal fluid (CSF) is reduced because of the formation of Lewy bodies for PD or PDD patients as compared to healthy subjects [13–16]. However, the reported results for the variations in the concentration of α-synuclein in blood are not consistent [17–20]. The main reason for the inconsistent assay results for plasma α-synuclein is the poor low-detection limit of assays. According to these reports [13–20], the enzyme-linked immunosorbent assay (ELISA) is currently used for assaying α-synuclein in either CSF or plasma. α-synuclein is expressed and is abundant in the brain and spinal cord, but occurs in very low amounts in the peripheral blood system. ELISA is not able to precisely detect the proteins at ultra-low concentrations, such as α-synuclein in plasma. Thus, CSF instead of plasma is better for the assay of α-synuclein in the bio-molecular diagnosis of PD or PDD using ELISA.

CSF is usually collected via lumbar puncher, which is high-risk and uncomfortable. The early-stage diagnosis by assaying α-synuclein in CSF is not widely accepted by the general population. Alternatively, blood is much easier to obtain in clinics. To do this, a high-sensitivity detection technology is required to achieve the assay of ultra-low α-synuclein in plasma.

Authors have developed an immunoassay technology, so-called immunomagnetic reduction (IMR), for quantitatively detecting bio-molecules at ultra-low concentrations, e.g. 1–10 pg/ml or lower [21, 22]. The main reason contributed to the ultra-high sensitivity of IMR is the utilization of antibody-functionalized magnetic nanoparticles. These magnetic nanoparticles are well dispersed in reagent and can catch target bio-molecules everywhere in a tested sample. Besides, due to the nano-scaled sizes of particles, the total binding area is extremely large. Hence, antibodies immobilized on the surfaces of magnetic nanoparticles are highly efficiently able to associate with target bio-molecules and result in an ultra-high sensitive immunoassay using IMR. It has been demonstrated IMR can be applied to assay ultra-low concentration β-amyloids and tau protein in human plasma [23–25]. A clear discrimination between healthy subjects and patients with mild cognition impairment due to Alzheimer’s disease was evidenced by assaying plasma β-amyloids and tau protein [26]. These results motivated us to investigate the feasibility of assaying ultra-low concentration α-synuclein in human plasma to achieve a bio-molecular diagnosis of PD or PDD, or to differentiate PD from PDD according to the plasma α-synuclein concentration. In this work, the reagent for assaying α-synuclein by utilizing IMR is prepared. The characterizations of the reagent and assaying α-synuclein are explored. For comparison, the assay characteristics for α-synuclein using ELISA are examined. Finally, the preliminary results for discriminating PD patients, PDD patients and healthy subjects by assaying plasma α-synuclein are reported. Although the cross sectional study done in this work cannot address the prediction of the development of PDD in PD, the results might point to the potential use of this method of measuring plasma α-synuclein in differentiating PD from PDD.

## Results and discussion

The mean value of the hydrodynamic diameters for the antibody-functionalized magnetic Fe_3_O_4_ nanoparticles was found to be 55.5 nm and the standard deviation of particle hydrodynamic diameters was 12.7 nm. By using scanning electronic microscope, the mean value of the diameters for the antibody-functionalized magnetic Fe_3_O_4_ nanoparticles was obtained as ~40 nm. The reagent is superparamagnetic with the saturated magnetization of 0.3 emu/g. According to a previously published paper [27], the numbers of antibody-functionalized nanoparticles in 1-ml reagent with 0.3 emu/g are around 10^13^. The total surface area of antibody-functionalized magnetic nanoparticles in 1-ml reagent is around 1000 cm^2^. In experiment, 80-μl reagent is used. The total surface area of antibody-functionalized magnetic nanoparticles in 80-μl reagent for each assay is around 80 cm^2^. As compared with a 96-well ELISA plate, the binding area between antibody and target bio-molecules for each well is 0.45 cm^2^. Thus, the binding area with IMR is almost 180 times larger than that of ELISA.

**Fig. 1:**
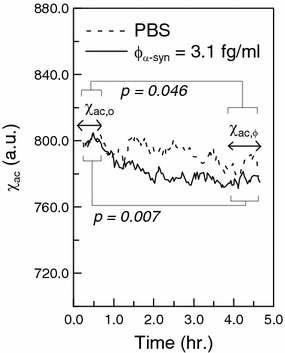
Bio-activity test for the antibody immobilized on magnetic Fe_3_O_4_ nanoparticles using immunomagnetic reduction

The bio-activity of the immobilized antibodies on magnetic nanoparticles is investigated by measuring the IMR signals due to the association between α-synuclein and antibodies on magnetic nanoparticles. The time dependent ac magnetic susceptibility χ_ac_ of reagent after mixing the reagent and the tested solution is recorded, as shown in Fig. 1 Two tested samples are prepared: one is pure PBS solution, the other is 3.1-fg/ml α-synuclein solution. The dashed line in Fig. 1 denotes the time dependent ac magnetic susceptibility χ_ac_ of the mixture of reagent and PBS solution. Clearly, temporal χ_ac_ with the dashed line almost remains unchanged. However, as to the solid line corresponding to the mixture of reagent and 3.1-fg/ml α-synuclein solution, the temporal χ_ac_ descends in 45 min and then reaches a lower level. A significant reduction in the ac magnetic susceptibility χ_ac_ of the reagent due to the association between α-synuclein and the antibodies on the magnetic nanoparticles is observed.

To quantify the reduction in the ac magnetic susceptibility χ_ac_ of the reagent, the initial/final χ_ac_ before/after the association between α-synuclein and antibodies on magnetic nanoparticles is calculated according to the temporal χ_ac_ shown in Fig. 1. As addressed in previously published papers [22, 28], the confidence intervals for the determination of reduction in ac magnetic susceptibility χ_ac_ of the reagent are that within the first and the last 40–50 min of the time dependent ac magnetic susceptibility χ_ac_ shown in Fig. 1. In this study, the data of ac magnetic susceptibility χ_ac_ of the reagent within the first and the last 45 min are used for determining the reduction in χ_ac_.

In Fig. 1, the *p* value for the ac magnetic susceptibility χ_ac_ between the intervals of the first and the last 45 min is found to be 0.046 for PBS solution. A slight reduction in the ac magnetic susceptibility χ_ac_ of reagent mixed with PBS is observed. As to 3.1-fg/ml α-synuclein solution, the *p*-value for the ac magnetic susceptibility χ_ac_ between the intervals of the first and the last 45 min is found to be 0.007. A clear reduction in the time dependent ac magnetic susceptibility χ_ac_ of reagent after being mixed with α-synuclein solution is evidenced.

The initial χ_ac_ is referred to as χ_ac,o_, which is the average value of χ_ac_’s within the first 45 min. The final χ_ac_ is referred to as χ_ac,φ_, which is the average value of χ_ac_’s within the last 45 min. The reduction in the ac magnetic susceptibility χ_ac_ of the reagent, e.g. IMR signal, is obtained via

1 $${\text{IMR }}\left( {{\% }} \right)\,{=}\,\left( {{{\upchi }}_{\text{ac,o}} - {{\upchi }}_{{{{{ac},\varphi }}}} } \right) / {{\upchi }}_{{ac,o}} {{ \times }}\; 1 0 0 {{\,\% }}$$

Via Eq. (1), the IMR signals for the dashed line and the solid line in Fig. 1 are calculated to be 1.56 and 2.13 %, respectively. The results shown in Fig. 1 reveal a background level for the IMR assay. Such a background level is mainly attributed to the electronic noises of the assay system. According to the duplicate measurements, the IMR signals for the PBS solution are 1.56 and 1.65 %. Thus, the background level of the IMR signal is 1.61 % with a standard deviation of 0.06 %.

**Fig. 2:**
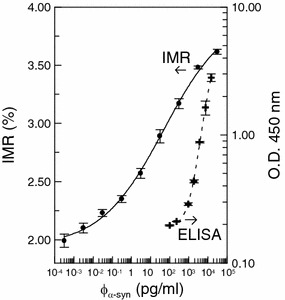
α-synuclein concentration dependent IMR signal (*solid line*) and optical absorbance density at 450 nm, O.D. 450 nm (*dashed line*)

The IMR signal as a function of the concentration of α-synuclein, i.e. IMR (%) − φ_α-syn_ curve, is plotted in Fig. 2. As the concentration of α-synuclein φ_α-syn_ increases from 3 × 10^−4^ pg/ml (=0.3 fg/ml), the IMR signal increases. The φ_α-syn_ dependent IMR (%) was found to follow the logistic function expressed as

2 $${\text{IMR}}\left( \% \right) = \;\frac{{{\text{A}} - {\text{B}}}}{{ 1 { + }\left( {\frac{{{{\varphi }}_{{{{\alpha - syn}}}} }}{{{{\varphi }}_{\text{o}} }}} \right)^{{{\gamma }}} }}\; + \;{\text{B}}$$

where A, B, φ_o_ and γ are fitting parameters. By fitting the data point in Fig. 2 to Eq. (2), the fitting parameters are obtained as A = 1.94, B = 3.95, φ_o_ = 49.7 and γ = 0.26. The fitting curve is plotted with the solid line in Fig. 2. The corresponding coefficient of determination R^2^ is 0.998. The fact R^2^ is very close to 1 implies φ_α-syn_ dependent IMR (%) is truly governed by the logistic function.

The parameter A in Eq. (2) is the value of IMR (%) as φ_α-syn_ extrapolates to zero. Usually, value A is a little higher than the background level. For example, A is 1.94 % and the background level here is 1.61 %. The difference between A and the background level is predominantly due to the noises caused by the dynamic equilibrium in the association between the protein molecules and the antibody-functional magnetic nanoparticles. However, A is not used as the low-detection limit. Conventionally, the low-detection limit is defined as the concentration showing an IMR signal higher than A by three times as the standard deviation of IMR signals for a low-concentration test, i.e. 3-σ criterion. In this experiment, the standard deviation of low-concentration tests is 0.028 %. Thus, the low-detection limit is the concentration having an IMR signal of 2.02 %. Via Eq. (2), the low-detection limit for assaying α-synuclein is found to be 0.3 fg/ml.

The α-synuclein concentration dependent optical absorbance density at 450 nm, O.D. 450 nm, using ELISA is plotted by cross symbols in Fig. 2. The experimental data are fitted to the logistic function

3 $${\text{O}} . {\text{D}} .\; 4 5 0 {\text{ nm = }}\frac{{{\text{A}}^{ '} {\text{ + B}}^{ '} }}{{ 1 { + }\left( {\frac{{{{\varphi }}_{{{{\alpha - syn}}}} }}{{{{\varphi }}_{\text{o}}^{ '} }}} \right){{\gamma }}^{ '} }}\;{ + }\;{\text{B}}^{ '}$$

The fitting parameters are found to be 0.189, 5.070, 13566.08 and 1.44 for A^′^, B^′^, φ_o_^′^ and γ^′^ in Eq. (3). The logistic function of Eq. (3) is plotted by the dashed line in Fig. 2. The coefficient of determination R^2^ between the cross symbols and the dashed line is 0.999. By utilizing the 3-σ criterion, the low-detection limit of assaying α-synuclein using ELISA is 79.04 pg/ml. It is obvious IMR is more sensitive than ELISA by a factor of 250,000 for assaying α-synuclein. As mentioned, the detecting sensitivity of IMR is higher than ELISA by a factor of 200 by taking the reacting surface into account. Additional factor of 1250 might be due to the ultra-low-noise magnetic sensor, i.e. high-T_c_ superconducting quantum interference device (SQUID) magnetometer. High-T_c_ SQUID magnetometer shows a noise level of 50 fT/Hz^1/2^, which is lower than the magnetic signal generated by a single magnetic nanoparticle by three orders of magnitude. This implies that the reduction in ac magnetic signal resulted from a single magnetic nanoparticle due to the associating with target bio-molecule can be sensed by high-T_c_ SQUID magnetometer. Hence, the ultra-low-noise high-T_c_ SQUID magnetometer is extremely sensitive to the reduction in ac magnetic signal of reagent and shows ultra-high sensitivity in assaying bio-molecules.

**Fig. 3:**
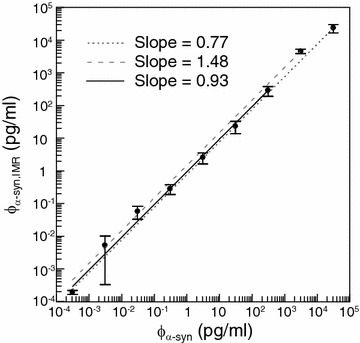
Converted α-synuclein concentration φ_α-syn,IMR_ versus spiked α-synuclein concentration φ_α-syn_ in PBS solution for the analysis of dynamic range for assaying α-synuclein

In addition to the low-detection limit, the dynamic range of assaying α-synuclein using IMR is an important characteristics. To examine the dynamic range, the experimental IMR signals in Fig. 2 are converted to concentrations of α-synuclein via Eq. (2). The converted concentrations of α-synuclein are denoted by φ_α-syn,IMR_. The correlation between φ_α-syn,IMR_ and φ_α-syn_ is examined, as shown in Fig. 3. In Fig. 3, the linearity between φ_α-syn,IMR_ and φ_α-syn_ can be obtained. According to the regulation issued by US Food and Drug Administration (FDA), the slope of the linearity in Fig. 3 must be between 0.90 and 1.10. In Fig. 3, if the φ_α-syn,IMR_’s for the α-synuclein concentration φ_α-syn_’s from 0.31 fg/ml to 31 ng/ml are used, the slope of the φ_α-syn,IMR_-φ_α-syn_ curve is 0.77 and the coefficient of determination R^2^ is 0.991, as plotted by the dotted line in Fig. 3. The slope of the dotted line in Fig. 3 does not meet the requirement of the US FDA. The concentration range of α-synuclein for investigating the assay dynamic range should be narrowed. Hence, the highest φ_α-syn,IMR_ in Fig. 3, i.e. with φ_α-syn_ being 31 ng/ml, is ignored. The linear curve between φ_α-syn,IMR_ and φ_α-syn_ within the range from 0.31 to 3.1 ng/ml is plotted by the dashed line in Fig. 3. The slope of the dashed line is 1.48 and the coefficient of determination R^2^ is 0.999. The slope of the dashed line is much higher than the requirement of the US FDA. It seems the second highest φ_α-syn,IMR_ in Fig. 3 should also be ignored. The linear curve between φ_α-syn,IMR_ and φ_α-syn_ within the range from 0.31 fg/ml to 310 pg/ml is plotted by the solid line in Fig. 3. The slope of the solid line is 0.93 and the coefficient of determination R^2^ is 0.999. Notably, the slope of the solid line meets the requirement of the US FDA. Thus, the dynamic range of α-synuclein concentration for IMR assay is from 0.3 fg/ml to 310 pg/ml.

**Fig. 4:**
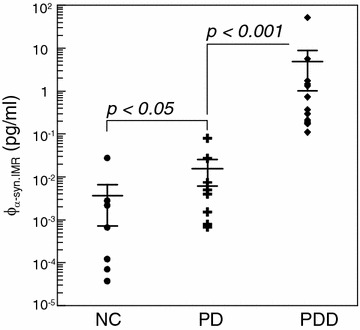
Detected plasma α-synuclein concentrations using IMR for healthy subjects, PD patients and PDD patients

The data shown in Fig. 2 prove the IMR assay is extremely sensitive and might possibly detect α-synuclein in human plasma. Plasma samples contributed by nine healthy persons, nine PD patients and fourteen PDD patients were collected for prior study on the discrimination between healthy subjects, PD patients and PDD patients by using IMR. The demographic information of the collected 33 subjects is listed in Table 1. The detected concentrations φ_α-syn,IMR_ of α-synuclein in human plasma are shown in Fig. 4. The plasma φ_α-syn,IMR_’s for PDD patients range from 0.1 to 100 pg/ml, while the plasma φ_α-syn,IMR_’s for healthy subjects are much lower than 0.1 pg/ml. The plasma φ_α-syn,IMR_’s for PD patients distribute between those of healthy subjects and PDD patients. The *p* value in terms of plasma φ_α-syn,IMR_ between healthy subjects and PD patients was found to be 0.005, which reveals the fact that PD patients show higher concentrations for plasma α-synuclein as compared to healthy subjects. In Fig. 4, a clear discrimination in plasma φ_α-syn,IMR_ between PD patients and PDD patients was observed (*p* < 0.001). According to the results in Fig. 4, the concentration plasma α-synuclein keeps raising as a healthy subject suffering from PD and progressing to PDD. It is worthy noting that the age is matched between healthy subjects and PD patients (*p* > 0.05), as well as between PD patients and PDD patients (*p* > 0.05).

**Table 1:** Demographic characteristics of the subjects

Group	Healthy subjects	PD with normal cognition	PDD patients
Numbers	9	9	14
Female/Male	4/5	4/5	7/7
Age (years)	38–73	38–85	60–81
MMSE (mean ± SD)	29.0 ± 1.1	28.7 ± 1.2	18.7 ± 6.3
Disease duration (years)	–	9.3 ± 6.7	10.1 ± 5.3

*PD* Parkinson disease; *PDD* Parkinson disease dementia; *MMSE* mini-mental state examination; *SD* standard deviation

Previous studies have shown that α-synuclein would be released from neurons by exocytosis into body fluids, including CSF and plasma, which contributes to cell-to-cell transmission of α-synuclein pathology in the brain [29]. Numerous studies have focused on checking levels of either total or oligomeric α-synuclein in plasma samples from patients with PD compared with healthy controls but the results are conflicting [30]. Since phosphorylated and fibrillar α-synuclein are the main pathological forms of the protein, one recent study observed that plasma level of phospho-α-synuclein was higher in early-stage PD samples without dementia than controls [31]. These observations suggest the feasibility and potentiality of plasma level of α-synuclein (either total, oligomeric or phosphorylated form) could partly reflect the α-synuclein pathology in the brains of PD patients. Furthermore, cortical Lewy body/neuritic pathology is more extensive in PDD than in PD without dementia, which implies the α-synuclein burden in plasma is more severe in PDD than in PD. Our results supported this hypothesis that plasma level of α-synuclein is significantly higher in PDD than in PD with normal cognition, which level is slightly higher than healthy controls. As amyloid β plaques and tau neurofibrillary tangles, the hallmark pathologies of Alzheimer’s dementia, are also observed and correlate with cognitive status in patients with PDD [29], future studies incorporating assessing phospho-α-synuclein, amyloid β protein, total and phospho-tau in plasma levels of PDD are needed to better understanding the pathophysiology of PDD.

In plasma samples, heterophilic antibody is a major confounder and interferes the assaying results by sandwich ELISA method [32]. Heterophilic antibody (HA) is defined as one of the common interference materials for immunoassay according to the guidance of Clinical and Laboratory Standards Institute (CLSI-EP-A2: Interference Testing of Clinical Chemistry) [33]. IMR method showed low-interference and high-specificity effects in comparison with ELISA through previous researches [34–36]. The selection mechanism is based on centrifugation force contributed from oscillating magnetic nanoparticles in reagent. The details have been discussed in previous research [37]. In fact, not only HA but also naturally existed biomolecules of frequently used drug in plasma are prevented from associating with magnetic nanoparticles via the selection mechanism [36]. This features IMR a high-specificity methodology for clinical analysis of plasma biomarkers of Parkison’s disease.

Clinically, patients first are diagnosed with PD and in later stages of the disease may develop dementia and thus get the diagnosis of PDD; Hence, biomarkers that can predict or diagnose early stages of progression to PDD in PD subject would indeed be of clinical significance. According to the results in Fig. 4, the plasma α-synuclein in PDD patients show clearly higher level than that in PD patients (*p* < 0.001). This implies that plasma α-synuclein is promisingly used as a clinical parameter monitoring the progression to PDD in PD patients.

## Conclusions

By immobilizing antibodies against α-synuclein onto magnetic nanoparticles, the reagent for assaying α-synuclein is developed. Through utilizing immunomagnetic reduction (IMR) with aid of high-T_c_ SQUID magnetometer, the dynamic range of assaying α-synuclein is from 0.3 fg/ml to 310 pg/ml. The ultra-sensitivity SQUID-based IMR is applied to assay human plasma α-synuclein. The preliminary results show a clear difference in the concentrations of plasma α-synuclein between healthy subjects, PD patients and PDD patients. This method seems promising to apply IMR to diagnosis of PD and PDD by assaying plasma α-synuclein.

## Method

The reagent for assaying α-synuclein consists of magnetic Fe_3_O_4_ nanoparticles (MF-DEX-0060, MagQu) functionalized monoclonal antibodies (sc-12767, Santa Crusz Biotech.) against α-synuclein. The detailed processes for immobilizing antibodies onto magnetic Fe_3_O_4_ nanoparticles are discussed in References [38, 39]. The antibody-functionalized magnetic Fe_3_O_4_ nanoparticles are dispersed in pH-7.2 phosphate-buffered saline (PBS) solution. The distribution of particle diameters is analyzed by dynamic laser scattering (Nanotrac-150, Microtrac). The magnetic concentration of reagent is measured using a vibrating sample magnetometer (HysterMag, MagQu). The bio-activity of the antibodies on the magnetic nanoparticles is examined by an IMR analyzer (XacPro-S, MagQu). The IMR analyzer is an ac magnetosusceptometer equipped with a high-T_c_ superconducting-quantum-interference-device (SQUID) magnetometer as a magnetic sensor. The details of the ac magnetosusceptometer are described in References [23, 40]. To establish the relationship between the IMR signal and the concentration of α-synuclein, α-synuclein (ab51189, Abcam) spiked in PBS solutions is prepared. For each measurement of the IMR signal, 80-μl reagent is mixed with 40-μl α-synuclein solution, followed by detection of the IMR signal using an IMR analyzer (XacPro-S, MagQu). Duplicate measurements are performed for IMR signals with each concentration of α-synuclein solution. In addition to the measurements of the IMR signals, a commercial ELISA kit (KHB0061, Novex) is applied to find the α-synuclein concentration dependent optical absorbance unit.

Volunteers participating in this study were given a medical checklist of major systemic diseases, operations and hospitalizations. Volunteers reporting uncontrolled medical conditions including heart failure, recent myocardial infarction (in the past 6 months), malignancy (in the past 2 years), or poorly controlled diabetes (HbA1C > 8.5) were excluded. Volunteers also received physical examinations. Eight healthy subjects and six patients with PD were enrolled in this study. The study was approved by the ethics committee and institute review board of the university hospital.

Participants were asked to provide a 10-ml non-fasting venous blood sample (K3 EDTA, lavender-top tube). Each sample was assigned a registry number following the sampling sequence; hence, colleagues in the laboratory were blind to the clinical status and the demographic data of the subjects. The blood samples were centrifuged (2500*g* for 15 min) within 1 h of collection and the plasma was aliquoted into cryotubes and stored at −80 °C for less than three months until being thawed for measurement via IMR. 80-μl of reagent was mixed with 40-μl of plasma for the measurement of α-synuclein concentration via IMR. Duplicate measurements were performed for each plasma sample.

Nine human plasma samples from healthy subjects aged from 38 to 73 years, 9 human plasma samples from PD patients (38–85 years old) and 14 human plasma samples from patients with PDD (60–81 years old) were used for the α-synuclein assay using IMR. PD and PDD patients were identified using clinical symptoms. It is worthy noting that PD patients are cognitively normal. All of the enrolled patients provided informed consent before undergoing the procedure and this study was approved by National Taiwan University Hospital Research Ethics Committee.
